# Retraction: Enhancing the decision optimization of interaction design in sustainable healthcare with improved artificial bee colony algorithm and generative artificial intelligence

**DOI:** 10.1371/journal.pone.0352925

**Published:** 2026-07-08

**Authors:** The PLOS One Editors

The *PLOS One* Editors retract this article [1] because it was identified as one of a series of submissions for which we have concerns about potential manipulation of the publication process. These concerns call into question the validity and provenance of the reported results. We regret that the issues were not identified prior to the article’s publication.

In addition, the article cited as Reference 28 was retracted before publication of [1].

All authors did not agree with the retraction.
